# Supplementary material for: Binding to nucleosome poises human SIRT6 for histone H3 deacetylation
Source: eLife. 2024 Feb 28;12:RP87989. doi: 10.7554/eLife.87989 (PMC10942634; doi:10.7554/eLife.87989)
Supplement: Supplementary file 1. — (a) Cryo-EM data collection, refinement and validation statistics. (b) Frequency of conformational clusters for each MD ensemble. Clusters featuring the H3 tail protruding between the DNA and the octamer are marked by a star. [file elife-87989-supp1.docx]

|  | SIRT6-nucleosome #1  (EMDB-16843,  EMDB-18497) | SIRT6-nucleosome #2  (EMDB-16842) | Composite map  (EMDB-16845)  (PDB 8OF4) |
| --- | --- | --- | --- |
| **Data collection and processing** |  |  | SIRT6 Rossmann fold:  Dataset #1 |
| Magnification | 81,000 | 270,000 | Nucleosome and SIRT6 Zn-finger: |
| Voltage (kV) | 300 | 300 | Dataset #2 |
| Electron exposure (e–/Å^2^) | 52 | 54.5 |  |
| Defocus range (μm) | 1.2-3.4 | 0.8-2.6 |  |
| Pixel size (Å) | 0.862 | 0.458 |  |
| Symmetry imposed | C1 | C1 |  |
| Initial particle images (no.) | 3,801,637 | 2,033,169 |  |
| Final particle images (no.) | 53,224 | 439,796 |  |
| Map resolution (Å)  FSC threshold | 3.6  0.143 | 2.94  0.143 |  |
| Map resolution range (Å) | 3.2-6.0 | 2.5-8.0 |  |
| **Refinement** |  |  |  |
| Initial model used (PDB code) |  |  | 3LZ0, 5X16 |
| Model composition  Non-hydrogen atoms  Protein residues  DNA residues  Ligands |  |  | 14,094  1,029  290  Zn: 1 |
| R.m.s. deviations  Bond lengths (Å)  Bond angles (°) |  |  | 0.013  2.040 |
| Validation  MolProbity score  Clashscore  Poor rotamers (%)  EMRinger score  Q-Score |  |  | 0.79  0.13  0.58  3.16  0.446 |
| Ramachandran plot  Favored (%)  Allowed (%)  Disallowed (%) |  |  | 96.43  3.57  0.0 |

**Supplementary File 1a| Cryo-EM data collection, refinement and validation statistics.**

| Cluster number | MD1 (5µs) | MD2 (5µs) | MD3 (5µs) |
| --- | --- | --- | --- |
| **With SIRT6** |  |  |  |
| 1 | 32 % | 56 % | 37 % * |
| 2 | 23 % | 11 % | 11 % |
| 3 | 16 % | 6 % * | 10 % * |
| 4 | 7 % | 5 % * | 10 % * |
| 5 | 6 % * | 5 % * | 9 % * |
| 6 | 5 % | 5 % * | 7 % |
| 7 | 5 % * | 4 % * | 7 % |
| 8 | 3 % | 4 % * | 5 % * |
| 9 | 2 % | 3 % * | 3 % |
| 10 | 1 % | 1 % * | 1 % |
| **Without SIRT6 (control)** |  |  |  |
| 1 | 34 % | 37 % | 34 % |
| 2 | 22 % | 17 % | 22 % |
| 3 | 9 % | 15 % | 16 % |
| 4 | 8 % | 11 % | 9 % |
| 5 | 8 % | 6 % | 8 % |
| 6 | 8 % | 4 % | 5 % |
| 7 | 5 % | 3 % | 4 % |
| 8 | 3 % | 3 % | 2 % |
| 9 | 2 % | 2 % | <1 % |
| 10 | 1 % | 2 % | <1 % |

**Supplementary File 1b | Frequency of conformational clusters for each MD ensemble.**

Clusters featuring the H3 tail protruding between the DNA and the octamer are marked by a star.
